# From Microscopy to Nanoscopy: Contemporary Physical Methods in Mitochondrial Structural Biology

**DOI:** 10.3390/ijms27052361

**Published:** 2026-03-03

**Authors:** Semen V. Nesterov, Anton G. Rogov, Raif G. Vasilov

**Affiliations:** National Research Center “Kurchatov Institute”, Akademika Kurchatova pl. 1, 123182 Moscow, Russia; rogov_ag@nrcki.ru (A.G.R.); vasilov_rg@nrcki.ru (R.G.V.)

**Keywords:** mitochondria, bioenergetics, mitochondrial morphology, super-resolution, cryo-electron tomography, fluorescent markers, spectroscopy

## Abstract

Mitochondria play a crucial role in cellular bioenergetics, signaling, and metabolism; yet, many fundamental mechanisms such as the proton transfer along the membranes, the link between membrane curvature and oxidative phosphorylation, and the nanoscale organization of enzyme supercomplexes remain poorly understood due to the limitations of classical biochemical approaches. This review addresses this gap by systematically analyzing the contemporary physical methods used to investigate the mitochondrial structure and function from the micro to nano scale. It covers advanced fluorescence and super-resolution microscopy, electron and volume electron microscopy, and scanning probe techniques, as well as cryo-electron tomography for resolving supramolecular assemblies in near-native conditions. The review highlights the applications of the modern fluorescent probes, expansion and phase microscopy, and machine-learning-based image analysis for a quantitative assessment of the mitochondrial morphology, membrane potential, and dynamics in living cells and tissues. Complementary spectroscopic and scattering methods, including Raman spectroscopy, NMR, and X-ray and neutron scattering, are discussed as tools for probing the redox state, metabolite composition, and membrane organization. Emphasis is placed on integrating high-resolution experimental data with advanced computational frameworks to test competing models of mitochondrial function and pathology, and to guide the development of biomimetic and biomedical technologies.

## 1. Introduction

Biological science at all stages of its development has been fundamentally based on physical and chemical methods of investigation. This is particularly evident in the fields of molecular biology and its specialized branch, bioenergetics. In the 20th century, biochemical methods enabled significant progress in understanding cellular metabolism, including the identification of all key metabolic processes occurring within mitochondria. However, biochemical methods alone are insufficient to provide detailed information about molecular-level processes and the structural organization of living systems. For this purpose, scientists have an extensive arsenal of physical measurement techniques that have been actively adapted over recent decades for studying biological objects, in some cases allowing data acquisition not only in vitro but also in situ and even in vivo.

In silico methods, enabling the highly accurate modeling of processes based on empirical data or first principles (ab initio), are also rapidly developing. Computational methods are most effective when combined with experiments and are sometimes essential tools for analyzing complex experimental data. For example, the advanced computer processing of two-dimensional images and three-dimensional models, including machine-learning algorithms, has become an integral part of modern microscopy techniques.

Describing all modern physical methods applied across the entire range of biological objects in a single review is challenging to do even superficially; therefore, this work focuses on the capabilities of contemporary physical methods applied solely to mitochondria. These double-membrane organelles, acting as key centers of energy production, metabolism, and signaling in all eukaryotes, have attracted increasing scientific interest in recent years. They are especially intriguing from the perspective of physical processes due to the presence of strong electric fields (a potential difference of 180 mV across a membrane thickness of approximately 5 nm corresponds to an electric field of 36 million V/m), and the working principles of mitochondria present promising targets for the reverse-engineering of living systems aimed at developing biomimetic technological processes, including improvements in fuel cells, supercapacitors, and biosensors technologies.

While the chemiosmotic theory by P. Mitchell, formulated in the 1960s, remains the classical explanation of mitochondrial function, many aspects of the operation and regulation of mitochondrial enzyme complexes remain incompletely understood. Recent data suggest that the accumulation and transfer of electrical charges in the form of protons predominantly occur at the interphase boundary of the branched inner membrane (see reviews [1,2]), effectively making mitochondria proton-based supercapacitors. Other authors emphasize the significance of polarization at this interphase boundary, comparing mitochondria to electrets functioning on protons [3]. Recent findings also indicate a possible direct involvement of potassium ions in the operation of mitochondrial ATP synthase [4]. New data showing mitochondrial heating to temperatures exceeding the surrounding cytosol by more than 10–15 K [5] raise questions about heat dissipation mechanisms and the incorporation of temperature gradients into existing theories of mitochondrial function.

Therefore, the necessity for developing new or refining existing physical methods to study mitochondria is evident. These methods should complement experimental data with a high spatial and temporal resolution, elucidating the details of mitochondrial function that have so far been inaccessible for direct measurement, thus hindering the verification or falsification of existing scientific hypotheses.

This review systematizes the information on the investigation of various aspects of the mitochondrial structure and function using physical research methods such as different types of microscopy and spectroscopy, spin resonance, and various types of the elastic and inelastic scattering of particles (neutrons, photons, and electrons). Using characteristic experimental examples involving mitochondria or their fragments (membranes, proteins, and complexes), the review indicates the mitochondrial processes that each method can analyze and outlines general directions for the further development of these studies applied to mitochondria.

## 2. Fluorescence-Based Microscopy of Mitochondria

Classical optical microscopy is poorly suited for studying mitochondria. When studying whole cells, it is impossible to visually identify the location of these organelles, and, in isolated mitochondrial preparations, their sizes are generally on the order of a micrometer in diameter, which is comparable to the wavelength of visible light, resulting in a limited information yield from such studies. Consequently, the most popular approach is using optical microscopy methods combined with dyes, often fluorescent ones.

### 2.1. Fluorescent Microscopy of Mitochondria

The mitochondrial morphology and membrane potential represent integrative indicators of the organelle’s functional state and are inseparably linked to the bioenergetic characteristics of cells. A quantitative assessment of these parameters by fluorescent microscopy provides unique opportunities for studying the mechanisms of cellular adaptation to metabolic challenges, pathogenesis of diseases associated with mitochondrial dysfunction, and therapeutic efficacy [6,7,8]. The mitochondrial morphology not only reflects the metabolic status but is also involved in regulating cellular bioenergetic parameters. Some systems show a direct quantitative correlation between the morphological indicators and oxidative metabolism functional parameters of cells [9,10]. The magnitude of the mitochondrial membrane potential usually correlates with their morphology as well [11].

Widefield and confocal microscopy enable the real-time assessment of changes in mitochondrial morphology and membrane potential under various physiological and pathological stimuli. Within timescales from hundreds of milliseconds to minutes, rapid transitional processes such as local potential changes in response to depolarizing agents (e.g., FCCP), ATP synthase modulators (e.g., oligomycin), or respiratory inhibitors can be detected [8,12], as well as mitochondrial morphological changes under oxidative stress [13,14,15,16,17,18,19], various pathologies [7,20], and aging [21].

A critical source of variability in morphometric measurements is the choice of fluorescent marker. Over the recent decades, the array of available probes has significantly expanded—from classical rhodamine derivatives to innovative Si-rhodamine constructs with cyclo-octatetraene (COT) triplet state quenchers. Each dye has a unique set of advantages and limitations, making the choice of an optimal probe critically important for the success of a particular experiment (see Table 1 and Section 2.2 below).

### 2.2. Fluorescent Probes for Mitochondrial Studies

#### 2.2.1. TMRM and TMRE

Tetramethylrhodamine methyl ester (TMRM) and ethyl ester (TMRE) are lipophilic cationic dyes specifically designed for the quantitative assessment of the mitochondrial membrane potential [22,23,24]. At a typical resting potential of −60 mV, the intracellular concentration of these probes is approximately tenfold higher than the extracellular; at the mitochondrial membrane potential of around −180 mV, very high concentrations accumulate in the matrix, rendering the mitochondria intensely fluorescent [25,26,27,28,29]. The principal limitation of TMRM and TMRE is their washout from cells upon the loss of the mitochondrial potential, restricting their use in fixation experiments or treatments affecting the mitochondrial energetic state [24,29]. Additionally, rhodamine dyes are susceptible to both photoinduction and photobleaching depending on the experimental conditions, necessitating the minimization of light exposure during their use [23].

#### 2.2.2. Rhodamine 123

Rhodamine 123 is a cell-permeable cationic fluorescent dye sequestered by active mitochondria. It demonstrates low cytotoxicity at appropriate concentrations and is commercially accessible. Unlike lipophilic rhodamine and carbocyanine dyes, Rhodamine 123 appears not to stain the endoplasmic reticulum and is rapidly taken up by cells from the incubation medium. However, it is highly sensitive to photobleaching and exhibits a pronounced photoinduced toxicity. Concentrations above 1 μM can interfere with mitochondrial respiration and ATPase activity, while those exceeding 10 μM induce mitochondrial swelling [30]. Its fluorescence depends nonlinearly on the mitochondrial membrane potential, making it more suitable as a qualitative rather than quantitative probe [31].

#### 2.2.3. MitoTracker Red CMXRos

MitoTracker Red CMXRos covalently binds to thiol groups of mitochondrial proteins and lipids, distinguishing it from other mitochondrial probes by allowing fluorescent labeling to endure aldehyde fixation and permeabilization by detergents [30]. However, its accumulation depends on both the membrane potential for the initial uptake and covalent binding to mitochondrial proteins and peptides, which may lead to the misinterpretation of the fluorescence intensity changes as direct indicators of the mitochondrial potential [32].

#### 2.2.4. MitoTracker Green FM and Deep Red FM

MitoTracker Green FM and Deep Red FM are carbocyanine dyes incorporating a cell-retentive moiety into their chemical structure. Unlike rhodamine variants, they lack a thiol-reactive chloromethyl group and exhibit potential-independent staining [33], although some studies report a strong correlation between dye accumulation and mitochondrial energization [30,34]. MitoTracker Green FM is widely used due to its availability in green fluorescence channels and facilitates the visualization of the mitochondrial morphology independent of the functional state. However, it may accumulate in other organelles and does not reliably correlate with the mitochondrial metabolism [34]. The primary advantage of MitoTracker Deep Red FM is its far-red emission, making it suitable for multiplexing with blue and green probes and deep tissue imaging [33].

#### 2.2.5. JC-1

The ratiometric probe JC-1 has become widely employed for the microscopic and cytometric assessment of the mitochondrial membrane potential due to its ability to accumulate in energized mitochondria and form spectrally distinct J-aggregates separate from monomers [31,35]. The monomer–aggregate system is highly sensitive and responds linearly to mitochondrial depolarization. Moreover, JC-1 can be excited at 405 nm, enhancing the linearity of fluorescence spectral changes relative to the membrane potential magnitude [36]. Nevertheless, JC-1 is prone to photobleaching, limiting its use in time-lapse studies, and the local formation of J-aggregates within mitochondria complicates morphological assessment [30,31].

#### 2.2.6. Carbocyanine Dyes

DIOC2(3) and other carbocyanine family dyes are highly potent potential-dependent fluorescent probes for the real-time assessment of mitochondrial function, exhibiting spectral shifts depending on the mitochondrial dye concentration [37]. Equilibrium of the emission spectra is achieved faster than for JC-1 [23], but high dye concentrations inhibit respiratory chain activity and induce mitochondrial dysfunction [38].

#### 2.2.7. MitoView 633

MitoView 633 is a far-red fluorescent dye that accumulates electrophoretically in mitochondria, making it a promising candidate for bioenergetic studies. Systematic evaluations showed advantages over TMRM, including a reduced photobleaching rate and enhanced thermal stability, with far-red fluorescence allowing the co-use with red dyes [39].

#### 2.2.8. HBmito Crimson

HBmito Crimson is a novel Si-rhodamine-based fluorescent dye characterized by exceptional photostability, targeted accumulation on the inner mitochondrial membrane, and emission only upon binding to the inner membrane [40,41,42]. It is weakly cytotoxic, non-fluorescent in aqueous solutions, but becomes strongly fluorescent upon lipid membrane binding—a fluorogenic behavior that significantly reduces the background signal [40,43].

#### 2.2.9. PKMO (COT-Cy3)

PKMO (COT-Cy3) is a cyanine dye conjugated to cyclo-octatetraene (COT), a strategy developed to minimize photodamage by quenching triplet states. This conceptual advance introduces triplet-depleted dyes into STED nanoscopy to minimize photodamage [44].

#### 2.2.10. MitoSOX Red

MitoSOX Red (mitochondrial superoxide indicator) is a derivative of hydroethidine (dihydroethidium) specifically designed for the detection and quantification of mitochondrial superoxide (O_2_^−^). Its cationic triphenylphosphonium substituent provides an electrophoretically driven uptake into actively respiring mitochondria [45,46]. The main advantage is the ability to detect oxidative stress specifically in mitochondria [16], though limitations include the incomplete specificity to superoxide, the inability to assess antioxidant effects modifying the mitochondrial membrane potential, and the fluorescence dependence on the mitochondrial nucleic acid content [45,47]. Consequently, other mitochondria-targeted probes have been developed for the detection of various reactive oxygen species (ROS). MitoAR and MitoHR primarily react with hydroxyl radicals and hypochlorous acid, respectively, while MitoPY1 selectively detects mitochondrial H_2_O_2_ [48,49].

#### 2.2.11. Mito-pH

The pH sensor Mito-pH, created by integrating a pH-sensitive FITC fluorophore with a pH-insensitive hemicyanine moiety, provides dual ratiometric pH measurement modes. A linear and reversible ratiometric response within pH 6.15–8.38 makes it ideal for the practical monitoring of mitochondrial pH fluctuations in living cells [50,51]. Another approach employs an M-pH probe based on hemicyanine with a stable π-electron conjugated merocyanine system. A lipophilic cationic benzyl group ensures mitochondrial accumulation, while the phenolic unit serves as a recognition element for ratiometric pH sensing [52].

#### 2.2.12. Limitations

It is important to remember that using fluorescent dyes is an invasive technique that may alter certain properties of the studied living system. This can result from specific interactions of the dye with proteins or lipids, as well as phototoxicity effects inherent to nearly all dyes. Additionally, high-intensity illumination in the microscope directly affects the sample, potentially causing local heating or free radical generation. Depending on the instrument settings, these effects can vary, complicating both the reproducibility and interpretation of the results. Common limitations of small molecule dyes also include multidrug resistance systems in microorganisms and P-glycoprotein expression in mammals [53,54]. When working with cell types expressing high P-gp levels (e.g., memory T cells, tumor cells, and hematopoietic stem cells), the inclusion of P-gp inhibitors (such as PSC833, verapamil, or cyclosporin H) during mitochondrial dye staining is recommended. Additional dye-independent methods (mtDNA content, mitochondrial volume, respiration assays, and proteomics) are necessary for the reliable assessment of mitochondrial features in these cells [53,54,55].

### 2.3. Genetically Encoded Biosensors

Genetically encoded calcium indicators (GECIs) have revolutionized the study of mitochondrial calcium signaling. The GCaMP family, based on circularly permuted GFP fused to calmodulin and the M13 domain, demonstrates an approximately 150% fluorescence increase upon Ca^2+^ binding. Progressive generations of GCaMP have been optimized for spectral properties, calcium affinity, brightness, and kinetics [56,57]. The GECO indicator family, derived from GCaMP3 via directed evolution, exhibits an improved signal-to-noise ratio and allows the simultaneous visualization of Ca^2+^ in multiple organelles or distinct compartments within a single organelle [56].

Real-time ATP dynamics visualization has been enabled by genetically encoded biosensors MaLions (Monitoring ATP Level intensiometric turn-on indicators), available in red, green, and blue variants targeted to the cytosol, mitochondria, and nucleus, respectively. These intensiometric sensors show a low pH sensitivity and facilitate the assessment of ATP dynamics and flux between compartments [58,59,60].

### 2.4. Confocal Microscopy

**Figure 1:**
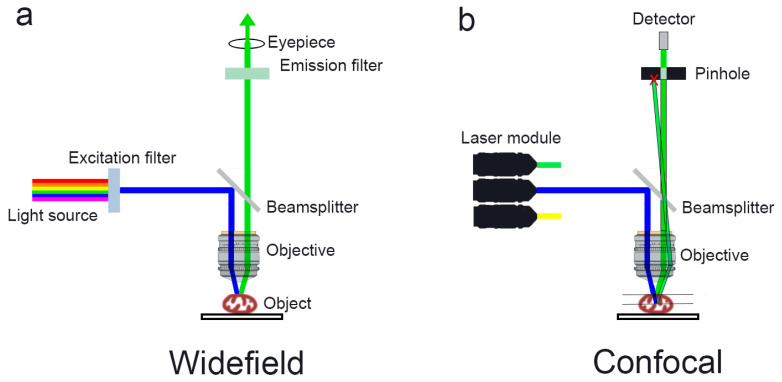
The operational principle of a fluorescent microscope: (**a**) widefield; and (**b**) scanning confocal.

Confocal microscopy operates on the principle of spatially aligning excitation and detection optical systems to effectively suppress scattered and out-of-focus emission (Figure 1) [61]. A laser beam focused by a high numerical aperture objective is concentrated to a single point within the sample; two-dimensional images are constructed by scanning the focus across the area of interest using scanning mirrors or a spinning disk with microlenses [62].

Confocal microscopy, utilizing point laser excitation, spatially conjugated aperture (pinhole), and precise scanning mechanisms, enables the following:

- A three-dimensional reconstruction of the mitochondrial architecture within the sample volume through z-stack acquisition;
- Enhanced optical contrast and spatial resolution;
- An efficient rejection of out-of-focus scattered light, thereby improving the signal-to-noise ratio;
- A greater penetration depth into biological tissue, albeit limited by light absorption, compared to widefield imaging modalities.

Both single-point scanning systems, which offer superior optical sectioning accuracy, and multi-point scanning platforms (e.g., spinning disk confocal systems), which simultaneously illuminate multiple spatial points to markedly increase the temporal resolution, are employed. The latter approach is particularly advantageous for capturing rapid mitochondrial morphological dynamics under conditions of cellular stress or therapeutic intervention [11,62,63].

### 2.5. Mathematical Postprocessing

For an objective assessment of mitochondrial clustering and the formation of mitochondrial networks, an important step is the mathematical processing of the obtained images. The key stages of such processing are briefly described below.

#### 2.5.1. Thresholding

A critical step in the analysis involves converting the grayscale confocal image into a binary mask, where background pixels are assigned a value of 0 and pixels corresponding to organelles are assigned a value of 1. Typically, the quality of the acquired image does not permit the use of classical image segmentation algorithms. Nevertheless, recent advances in machine-learning algorithms have facilitated the automation of the segmentation process, enabling the extraction of objective quantitative data from microscopy images [64,65,66,67].

#### 2.5.2. Connectivity Analysis and Network Skeletonization

Following the generation of the binary mask, a skeletonization procedure is applied, which compresses the mask components into zero-thickness lines while preserving the topological connectivity. A subsequent skeletal analysis (using the ‘analyze skeleton’ function in ImageJ v.1.5 or later) quantitatively assesses the number of branch junctions, branch lengths, and endpoints, thereby providing the metrics of complexity and connectivity of the network architecture [11].

#### 2.5.3. Validation and Quality Control

To ensure the robustness and reliability of the analysis, several quality control strategies are implemented [11,67]:

- A qualitative comparison of the binary mask with the original grayscale image to identify systematic over- or under-segmentation;
- A statistical evaluation of parameters using unbiased clustering algorithms (e.g., SPADE clustering) for the independent validation of the phenotypic classification;
- A correlation analysis between the independently measured parameters.

### 2.6. Fluorescence Lifetime Imaging Microscopy

Fluorescence Lifetime Imaging Microscopy (FLIM) constitutes a powerful tool for investigating the structural and functional organization of mitochondria at the cellular and subcellular levels. Unlike conventional intensity-based fluorescence microscopy methods, FLIM measures the duration a fluorophore remains in the excited state before returning to the ground state, providing measurements that are independent of the fluorophore concentration and photobleaching effects. This technology offers unique capabilities for the quantitative analysis of the mitochondrial metabolism, ultrastructure, and dynamics in living cells and tissues [68,69].

### 2.7. NADH-FLIM: Detection of Autofluorescence and NADH and FAD

NAD(P)H fluorescence lifetime imaging microscopy (NADH-FLIM) represents a noninvasive approach to assessing the energetic metabolism at the single-cell level. The method exploits the differences in fluorescence lifetimes between free NADH (~400 ps) and protein-bound NADH (~2500 ps). A longer average lifetime of NADH indicates a more oxidized redox state [70,71,72]. Two-photon FLIM microscopy (2P-FLIM) enables NADH measurements in deep tissue layers as a result of the excitation in the near-infrared spectral range. This technology has been successfully applied to studying neuronal cell differentiation, bone tissue metabolism assessment, and monitoring cancer cell responses to therapy [73,74].

### 2.8. FRAP and Photomanipulation Approaches

Fluorescence Recovery After Photobleaching (FRAP) remains a powerful technique for investigating the mitochondrial dynamics. FRAP experiments provide three types of information depending on the temporal scale of recovery: the mobility of the fluorescent molecule, mitochondrial continuity, and mitochondrial fission–fusion dynamics [56,75,76].

The enzyme-dependent FRAP (ED-FRAP) method for NADH enables the assessment of dehydrogenase activity in intact hearts. Optimized parameters allow reproducible measurements of NADH production rates under various metabolic disorders [76].

### 2.9. Two-Photon Microscopy for Deep Tissue Imaging

Two-photon microscopy (2PM) employs two low-energy photons in the near-infrared range to excite fluorophores, offering advantages including deep tissue penetration, spatially localized excitation, and extended observation times. The application of 2PM with adaptive optics (TPFM-AO) has enabled mitochondrial visualization through 85 μm of scattering bone marrow tissue in live mice at a 400 nm resolution [77].

Specialized two-photon mitochondrial probes exhibit a red-shifted emission. CMT-red demonstrates a high specificity and strong staining capacity for mitochondria, high photostability under two-photon excitation, and low cytotoxicity. This probe has been successfully used to visualize mitochondrial transport in primary cortical neurons and rat hippocampal slices at depths exceeding 200 μm [78].

### 2.10. Expansion Microscopy

Expansion Microscopy (ExM) represents an innovative super-resolution fluorescence microscopy approach that achieves a nanoscale resolution by physically enlarging biological samples embedded in a swellable polyacrylamide-based hydrogel. This method allows the visualization of subcellular structures, including the mitochondrial ultrastructure, using conventional fluorescence microscopes. The application of ExM to mitochondrial studies has uncovered new insights into the cristae morphology, protein localization, and mechanisms of mitochondrial dysfunction [79,80,81,82,83].

The method is based on the sequential steps of biomolecule anchoring to the hydrogel, polymerization, homogenization (disruption of cellular connections), and osmotic swelling in water. The expansion factor ranges from 4-fold [84] to greater than 21-fold for iterative three-dimensional expansion microscopy [85,86], directly enhancing the effective optical resolution [86,87].

Applied to mitochondria, ExM enables a detailed visualization of the inner membrane cristae morphology [81], the localization of key MICOS proteins [88], and dynamic structural changes under stress conditions [81,89]. ExM extends the capabilities of optical microscopy for studying the mitochondrial architecture and pathophysiology while preserving the three-dimensional spatial relationships of fluorescent markers [82].

The major advantages of ExM include its accessibility, versatility, and compatibility with thick tissues without requiring expensive super-resolution instruments [84,90,91]. The limitations encompass the inability to observe live cells [85], fluorescence loss [92], and potential anisotropic distortions during expansion [83].

Thus, expansion microscopy emerges as a potent tool for the nanoscale investigation of mitochondria and their roles in cellular physiology and pathology.

### 2.11. Structured Illumination Microscopy: Super-Resolution with Minimal Photodamage

**Figure 2:**
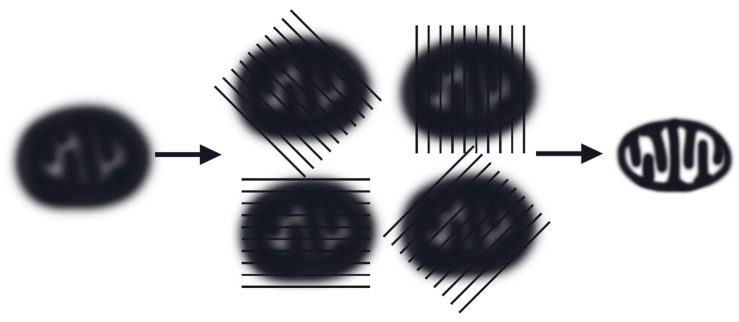
Principle of image acquisition using the SIM method.

Structured Illumination Microscopy (SIM) offers a resolution of approximately 100 nm and is considered the least phototoxic super-resolution technology. The SIM principle is based on the moiré effect—spatial modulation arising from the superposition of the high-frequency structure of the specimen with the high-frequency pattern of a known illumination template [93]. Technically, SIM employs a diffraction grating placed in the excitation laser beam path, generating a set of interfering beams (Figure 2) [40,94]. The zero-th and first diffraction orders form a sinusoidal illumination pattern of alternating light and dark stripes at the objective’s focal plane.

SIM provides a temporal resolution on the order of milliseconds and is successfully applied to visualizing the dynamics of the inner mitochondrial membrane and cristae, as well as to studying the interactions between organelles [95]. Although SIM does not always enable the distinction of individual cristae, this technique is indispensable for investigating groups of cristae and their interactions during mitochondrial dynamic processes [14,96,97].

### 2.12. STORM, PALM, and GSDIM: Superresolution Option for Fixed Samples

Stochastic Optical Reconstruction Microscopy (STORM) achieves a lateral resolution of 30 nm in live cells and up to 10 nm in fixed samples. Although STORM requires prolonged image acquisition and extensive post-processing, the method offers advantages at low illumination levels, making it suitable even for specific live-cell applications with a lower resolution and longer acquisition times than in fixed samples [98].

In addition to STORM, there are other methods from the single-molecule localization microscopy family that are suitable for mitochondrial research. Photoactivated Localization Microscopy (PALM) enables the super-resolution (~10–30 nm) imaging of mitochondrial proteins, nucleoids, cristae, and matrix structures in fixed cells using photoactivatable fluorescent proteins [99]. It excels at the precise localization of densely packed macromolecules within mitochondria (~200–500 nm), surpassing diffraction limits, though limited to fixed samples, specific photoactivatable fluorescent proteins, and thin sections or the total internal reflection fluorescence (TIRF) technique for optimal z-resolution [99].

Another interesting technique to surpass diffraction limit, suitable for use on fixed cell samples, is ground state depletion microscopy followed by individual molecule return (GSDIM). GSDIM can visualize mitochondria networks, uses standard organic dyes, is compatible with immunostaining, and can be correlated with higher-resolution tomography methods [100].

### 2.13. STED Microscopy: Visualization of Cristae Architecture

**Figure 3:**
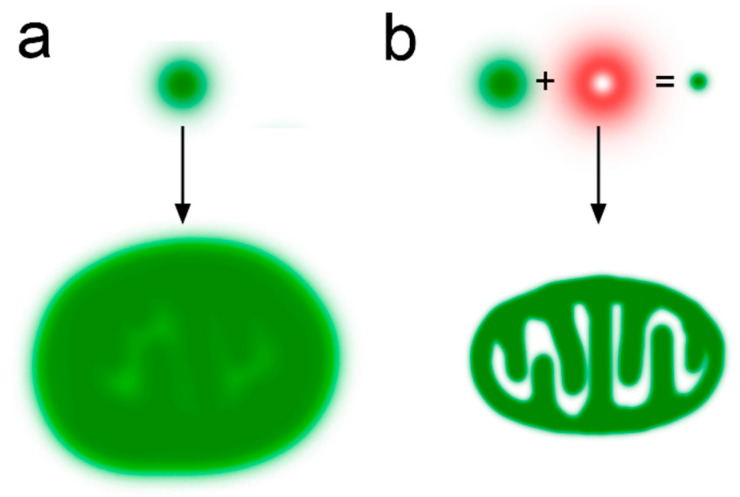
Principle of STED microscopy: (**a**) widefield fluorescence microscopy; and (**b**) STED microscopy.

Stimulated Emission Depletion (STED) microscopy is a powerful super-resolution technique enabling a spatial resolution of 40–50 nm in live cells. This method surpasses the diffraction limit of conventional light microscopy (200–250 nm) and allows the direct visualization of mitochondrial internal structures, including cristae [44,56,101,102]. The STED principle involves the illumination of the specimen not only with an excitation beam but also with a doughnut-shaped depletion beam that quenches the fluorophore fluorescence (Figure 3). The excitation beam position is known with a nanometer precision, allowing the determination of fluorophore molecule coordinates with enhanced accuracy.

The recent advances in STED microscopy are associated with the development of novel fluorescent probes exhibiting exceptional photostability, enabling the prolonged imaging of the inner mitochondrial membrane for over 50 min with a resolution of 35.2 nm [101]. The detailed visualization of the cristae dynamics has revealed ultrastructural changes in mitochondria during apoptosis, ferroptosis, and mitochondrial fission [40,42,43]. The application of STED microscopy has expanded to include the study of mitochondrial protein complexes. Multicolor STED imaging has allowed the simultaneous observation of MICOS (mitochondrial contact site and cristae organizing system) protein distribution, and interactions between mitochondria and the endoplasmic reticulum and cytoskeleton, as well as an investigation of cristae phenotypes in genetically modified cell lines [44,102].

### 2.14. MINFLUX: Nanometer Precision in Mitochondrial Biology

MINFLUX nanoscopy represents the most advanced super-resolution technology currently available [103]. The method combines the advantages of PALM/STORM and STED by employing a structured excitation beam with a zero-intensity center that is sequentially moved to the coordinates of individual fluorophores [104]. The iterative process involves determining the fluorophore position with a precision comparable to the beam size, shrinking the excitation field to one-third of the beam size, and repeating the search with smaller steps, achieving a localization precision of 3–5 nm in three-dimensional space [105]. Non-specific fluorescence and other background contributions require compensation procedures to yield reliable, unbiased localization estimates free from systematic errors. Modern MINFLUX systems implement real-time background compensation rather than post-processing.

MINFLUX has been successfully applied to studying the spatial distribution of MICOS complex subunits in human mitochondria. The Mic60 protein forms ring-like structures with diameters of 40–50 nm around individual cristae junctions. Mic19 is typically located in close proximity to Mic60, whereas the spatial coordination of Mic10 with Mic60 is less regular, indicating structural heterogeneity within MICOS [104].

3D MINFLUX can be used for mitochondrial visualization in brain tissue sections. This technology revealed a significant redistribution of the α-F1 subunit of ATP synthase in synaptic mitochondria [106], correlating with learning processes and synaptic plasticity. Additionally, a method combining MINFLUX with single-molecule fluorescence in situ hybridization (smFISH) has been developed to visualize individual mitochondrial mRNA molecules at a nanometer resolution [107].

Translocase of the outer mitochondrial membrane 20 (TOM20), a key component of the protein import complex, has been visualized in three-dimensional nanometer-scale clusters using GLF-MINFLUX (gradual labeling with fluorogenic probes for MINFLUX) [108].

The advantages of MINFLUX [109] are as follows:

- Molecular resolution: Achieving a 1–3 nm lateral resolution surpasses all other super-resolution methods by a factor of 5–100;
- Photon efficiency: It requires 20–100 times fewer photons than PALM/STORM for an equivalent localization accuracy;
- Sub-millisecond temporal resolution: The localization of a single fluorophore takes less than 5 microseconds, enabling tracking frequencies of up to 10 kHz, vastly exceeding camera-based methods;
- Isotropic 3D resolution: 2–3 nm precision in all three dimensions, unlike the anisotropic resolution in most other techniques;
- Multicolor imaging: There is a simultaneous visualization of multiple proteins with excitation wavelengths at 511, 560, and 647 nm;
- Live-cell compatibility: The low photon load permits studies in living cells without excessive photobleaching;
- Confocal background suppression: The combined scanning mode with a confocal diaphragm allows imaging depths up to 80 μm in tissue sections;
- Isotropic resolution in three dimensions [104].

The limitations are as follows:

- Complexity of the instrumentation: MINFLUX requires a highly sophisticated optical setup for 3D localization, spatial light modulators for donut beam formation, and active sample stabilization with a sub-nanometer precision [109];
- Sample and fluorophore requirements: Fluorophores must stochastically switch between bright and dark states [110];
- At a sub-nanometer resolution, fluorophores positioned closer than 10 nm (within the FRET range) may increasingly exhibit collective behavior, compromising the ability to resolve individual fluorophores;
- At a nanometer resolution, fluorescence distribution images may not always represent the precise location of the target biomolecules. The resolution achieved at the biomolecular level depends critically on the accuracy and completeness of molecular labeling. Fluorophore displacement from actual target proteins can be significant, often exceeding 10 nm in the case of indirect immunofluorescence labeling.

## 3. Interference and Phase Microscopy of Mitochondria

Techniques based on the phase measurement of light transmitted through a specimen allow imaging without the use of dyes. This approach enables the study of relatively thin and transparent biological structures and, with certain limitations, is applicable to isolated mitochondria as well as whole cells.

Over 20 years ago, dynamic phase microscopy demonstrated the capability to investigate mitochondria. It was revealed that the phase changes of light passing through mitochondria correlate with the transmembrane potential, accompanied by oscillations in phase thickness at frequencies of several Hertz [111]. Subsequent studies showed that mitochondrial swelling can be monitored by measuring the phase thickness [112]. Despite the initial interest, these approaches did not compete effectively with more precise and unambiguously interpretable methods, resulting in limited development.

**Figure 4:**
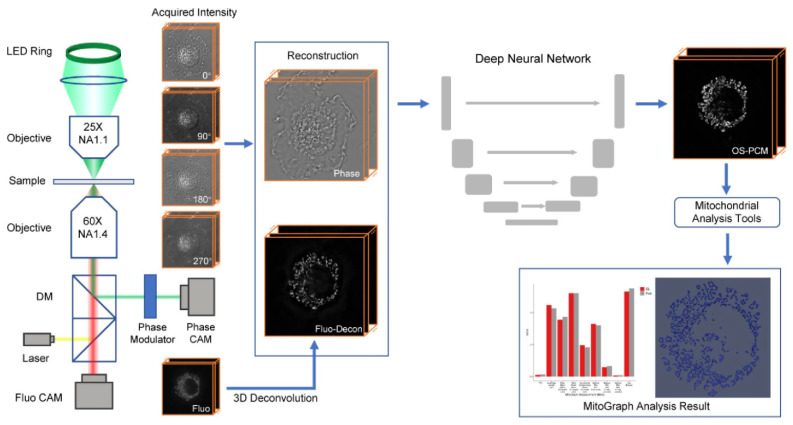
A general schematic diagram for image acquisition and processing to obtain information on mitochondrial networks using phase microscopy. The figure is adapted from the study [113].

However, recent advances propose a paradigm shift through the integration of combined technologies and data processing using convolutional neural networks [113,114]. It has become possible to identify mitochondria within intact cells on images acquired by high-resolution phase contrast microscopy following machine learning trained on fluorescently labeled mitochondrial images (Figure 4). This enables a quantitative analysis of the mitochondrial morphology and dynamics without artifacts related to staining and photobleaching. Studies indicate that this method achieves a high spatial and temporal resolution (approximately 270 nm and 250 frames per second), provides three-dimensional information on mitochondria and their networks across diverse cell types, and facilitates the analysis of dynamic processes such as mitochondrial fragmentation. Another promising application is the correlation of this method with fluorescence microscopy to assess the mitochondrial localization of fluorescently tagged proteins or compounds without additional mitochondrial dyes [115].

Interestingly, machine learning has been similarly applied to detecting mitochondria in cells using bright-field microscopy [116]. Thus, advances in machine-learning methods enable the extraction of significantly more information from the same datasets that previously did not allow the reliable identification of individual organelles. However, it should be noted that such approaches must be applied with caution, especially when analyzing samples that differ in any parameters from the dataset used for machine-learning training.

Therefore, the phase microscopy of mitochondria allows the assessment of their size characteristics and fluctuations in phase height, which indirectly reflect mitochondrial function. The use of machine learning has opened new possibilities for optical microscopy techniques, particularly phase microscopy, enabling the reconstruction of mitochondrial networks in cells without the use of specific dyes.

## 4. Transmission Electron Microscopy

### 4.1. TEM of Mitochondrial and Tissue Sections

One of the longest-established methods for the high-resolution structural investigation of mitochondria is transmission electron microscopy (TEM). These studies typically involve ultrathin sections of fixed (using aldehydes and osmium tetroxide) and contrasted (most commonly with uranyl acetate or lead citrate) isolated mitochondria or tissues.

The application of TEM to isolated and fixed mitochondria has enabled the study of volume changes and cristae structural remodeling under various functional states [117], osmotic swelling [118], and diverse pathologies such as hypoxia [119]. Due to the pivotal role of this method, it has significantly advanced the understanding of the mitochondrial structure and dynamics [120] and clarified the functions of many mitochondrial structural proteins. Consequently, electron microscopy swiftly became the gold standard in mitochondrial structural research. Given mitochondria’s characteristic double-membrane architecture with inner membrane folds, the manual identification of mitochondria in tissue sections is comparatively straightforward.

The method’s capabilities are further enhanced by its combination with antibodies targeted against proteins of interest. The use of secondary antibodies conjugated with metallic nanoparticles (the so-called immuno-electron microscopy technique) allows, with sufficient statistical sampling, an analysis of the protein distribution within organelles and the determination of the preferred subcompartmental localization of proteins within mitochondria [121,122].

In recent years, driven by the increasing standards of scientific publications and the necessity to detect subtle effects (e.g., early pathology stages, mutation impacts on the cristae ultrastructure, or assessments of test compounds), the problem of automating image processing and extracting statistically analyzable mitochondrial parameters has gained prominence [123,124]. This includes the development of machine-learning methods for detecting mitochondria in EM images of tissue sections [125]. The integration of new automation technologies may accelerate the workflow and enhance reliability compared to manual processing. An interesting enhancement in EM for improved convenience and performance is MultiCLEM, a high-throughput correlative light and electron microscopy technique that uses fluorescence barcodes to identify cell types in mixed populations and analyze the structure of organelles in them, including mitochondria [126].

Thus, the classical approaches to TEM of ultrathin sections enable the investigation of tissues, organelle contacts, and the collection of statistical data on membrane structures or antibody-labeled protein distributions across a large number of mitochondria. Therefore, in the near future, this method is unlikely to be fully replaced. A limitation of TEM is the paucity of volumetric structural information. This can be partially addressed by a tomography or analysis of serial sections from the same sample. However, scanning microscopy methods, which will be described later, are more specialized for studying three-dimensional structure.

### 4.2. Cryo-TEM and Mitochondrial Tomography

Cryogenic transmission electron microscopy (cryo-TEM) has become a new driver in structural biology. The most common methodology, called single-particle analysis, involves acquiring tens/hundreds of thousands of projections of the purified protein/complex by cryo-TEM with complex further computer processing leading to a 3D structure acquisition. The study of less stable complexes and supramolecular assemblies, whose interactions are disrupted by detergents—particularly complexes formed via lipid–protein interactions within membranes—is feasible only through minimally invasive methods that preserve native membranes, one of which is cryo-electron tomography (cryo-ET). It is made on thin sections (100–200 nm) of the tissue/mitochondria sample. Sample preparation for any cryo-TEM-based technique involves the rapid freezing of biological specimens (cells, organelles, membranes, and proteins) in liquid ethane cooled by liquid nitrogen temperature. This vitrification process prevents the formation of crystalline ice, preserving water in an amorphous state, and maintains the specimen in a near-native conformation compared to other fixation methods. The use of additional fixing or contrast agents is unnecessary, and all subsequent procedures are conducted at liquid nitrogen temperatures.

**Figure 5:**
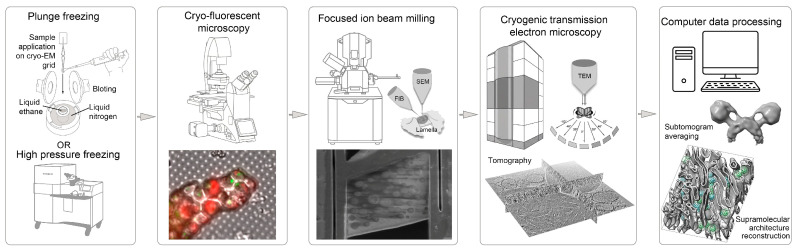
Cryo-TEM experiment protocol.

The simplified scheme of the cryo-TEM protocol is shown in Figure 5. Depending on the scientific question, after sample vitrification, an analysis via cryofluorescence microscopy can be performed to locate pre-introduced fluorescent labels or proteins. Subsequently, thin lamellae can be prepared by ion beam milling in a scanning microscope at regions of interest for further cryo-ET. This thinning step is essential for cells and large organelles, whereas it may be omitted for protein complexes or membrane preparations. For single-particle averaging tasks (e.g., protein complex studies), extensive image series are acquired by cryo-TEM. For cryo-ET, the sample is illuminated with a lower electron dose but imaged at multiple tilt angles, typically ranging from −60° to +60°.

An integral component of modern cryo-TEM protocols is the computational processing of acquired images. The ultimate goal is generally to obtain a three-dimensional model of the investigated proteins. A variety of algorithms and methodological approaches for this purpose are detailed in specialized reviews (e.g., [127]). The following sections provide examples of how cryo-TEM advances mitochondrial research.

Initially, it is important to note that mitochondrial complexes have been extensively studied using the single-particle approach. Particularly well-suited for this method are respiratory chain supercomplexes and ATP synthase dimers, whose structures have been described in dozens of publications. In summary, only cryo-TEM has enabled the acquisition of the complete structures of these proteins without relying on the hypothetical fitting of subunits and has permitted the refinement of certain structural features. A prominent example where cryo-TEM-derived structures markedly altered the understanding of protein function is ATP synthase, for which the X-ray structures of the membrane subunit were found to be non-native due to crystallization artifacts. The advent of cryo-TEM resolved the architecture of this crucial mitochondrial complex. A similar situation holds for respirasomes, where X-ray crystallography could not yield structures owing to the supercomplexes’ large size (several megadaltons, depending on the composition and organism). The implementation of both single-particle cryo-TEM and cryo-ET heralded a new era in the study of large mitochondrial complexes.

Conversely, while cryo-ET generally does not achieve the resolution of single-particle methods, it offers unique insights. Cryo-ET has demonstrated that ATP synthases not only dimerize but that their dimers assemble into oligomeric rows that deform the membrane, inducing a significant curvature [128,129,130]. Such findings could not be obtained using a single-particle analysis because that approach involves isolated protein complexes where dimer–dimer interactions are disrupted by detergents used in purification. Respirasomes, mitochondrial respiratory chain supercomplexes, share this membrane-bending property [131,132], facilitating the clustering of the oxidative phosphorylation system [133] under membrane pressure and macromolecular crowding [134]. Thus, the progress in cryo-ET has unveiled the mechanisms of the self-assembly and regulation of oxidative phosphorylation based on protein sorting according to their influence on the bilayer curvature.

Another notable application of cryo-ET is the analysis of whole mitochondria and the localization of matrix protein complexes. This approach has provided a direct visualization of the predicted contacts between ketoacid dehydrogenase complexes and mitochondrial Complex I by imaging their interactions within intact mitochondria and inner membrane preparations [135]. Absent cryo-ET, the demonstration of contacts between these proteins required more complex methodologies prone to artifacts. For example, the detection of interactions between fatty acid β-oxidation dehydrogenase complexes and Complex I involved co-precipitation assays via gel electrophoresis [136], the negative-stain electron microscopy of gels, and antibody labeling [137]. It is important to note that β-oxidation proteins are relatively small, complicating their cryo-ET detection, likely explaining the absence of further cryo-ET evidence for their association with Complex I in whole mitochondria.

An additional example of cryo-ET application to mitochondria is the characterization of supramolecular clusters of creatine kinases in the mitochondrial intermembrane space and the observation of their structural changes upon the disruption of the outer membrane integrity [138]. This highlights the importance of studies in native conditions.

Overall, the research on intact mitochondria using cryo-ET is rapidly advancing. The greatest progress has been achieved in unicellular algae, where mitochondria are less densely packed with protein, facilitating the better resolution of individual complexes and a higher resolution during subtomogram averaging. For instance, the spatial arrangement of ATP synthases in the mitochondrial membrane of *Polytomella* sp. has recently been determined, and subtomogram averages with a resolution sufficient to discern the rotational states of rotor subunits were obtained [139]. Subtomographic averaging involves processing similar to single-particle analysis, but for small tomogram volumes containing the protein of interest, rather than its two-dimensional projections. Due to a number of technical issues, this typically results in a lower resolution, but, with a sufficiently high initial tomogram quality and a large sample size, the resolution can rival that of other protein-structure-solving methods. Therefore, in *Chlamydomonas reinhardtii* mitochondria, the localization and visualization of respirasomes, ATP synthases, and several other large protein complexes have been achieved, with subtomogram averaging reaching resolutions better than 3 angstroms in intact mitochondria [140]. These studies mark a milestone in structural biology, bridging the gap between the organelle and molecular scales in structural investigations.

The examples above illustrate both the great potential of cryo-ET and underscore significant limitations, notably the challenge of primary protein identification, which intensifies as the protein size decreases. Therefore, although cryo-ET has opened new avenues for studying large supramolecular complexes, it remains limited in applicability for low-molecular-weight proteins (less than 100 kDa).

### 4.3. Scanning Transmission Electron Microscopy (STEM) 

An intriguing approach involves the integration of scanning and transmission electron microscopy within a single instrument, enabling the capture of nearly all electrons scattered by the specimen and thereby enhancing the analytical capabilities [141]. A key advantage of this setup for biological samples is the elimination of the need for heavy metal staining. The possibility of usage in cryogenic mode [142] gives this method an advantage against volume microscopy methods relying on chemical fixation, which can disturb the native structure [143]. A technical challenge of this method is the need for preliminary cell unroofing—the partial removal of the membrane to expose cytoplasmic structures. The subsequent exposure of deep layers can be achieved by sublimation under controlled temperature cycling in the cryo-STEM device allowing us to reduce artifacts associated with the deformation of native structures [144].

This method allows the visualization of organelles within intact cells at 3D resolutions on the order of a few nanometers and a field of view spanning several micrometers, with sample thicknesses around 1 μm [145]. Although promising for contextualizing mitochondria—including their cristae and large protein complexes—within whole cells, Cryo-STEM lacks the resolution needed for detailed protein studies compared to cryo-electron tomography. While it surpasses conventional transmission electron microscopy in a volumetric scope, it is outperformed by the other scanning microscopy techniques described below.

## 5. Scanning Electron Microscopy (SEM)

SEM has been employed to study mitochondrial dynamics, such as organelle fission and fusion and cristae morphological alterations under pathological conditions [146].

### 5.1. SEM of Freeze-Fractured Samples

SEM, a longstanding technique, substantially expanded the understanding of the mitochondrial three-dimensional architecture. Early protocols typically involved sectioning or fracturing tissue and isolated mitochondria samples at liquid nitrogen temperatures. This method yields valuable data on the mitochondrial 3D structure [147] and spatial distribution within cells [148]. Although cryogenic freezing is involved in preparative steps, fixation—similar to that in TEM—is performed prior to freezing [147]. SEM preparative protocols often use organic solvents (DMSO and ethanol), drying, osmium vapor treatment [149], or metal coating (the latter forming a protective conductive layer) [147]. Sample fracturing occurs at liquid nitrogen temperature; subsequent processing and analysis take place at room temperature. Therefore, as with classical TEM, samples examined by SEM cannot be considered native, since structural alterations or damage during preparation cannot be excluded. In this regard, these methods are significantly less native than modern cryo-electron microscopy protocols.

Cryo-SEM, a variant involving the cryo-fixation of hydrated samples, also exists [150]. However, more recent approaches enabling large-volume data acquisition—termed volume electron microscopy—have gained prevalence. Novel volumetric microscopy techniques allow the investigation of entire tissue volumes measuring hundreds of microns [151].

### 5.2. Focused Ion Beam (FIB-SEM) and Serial Block Face (SBF-SEM) Scanning Electron Microscopy

**Figure 6:**
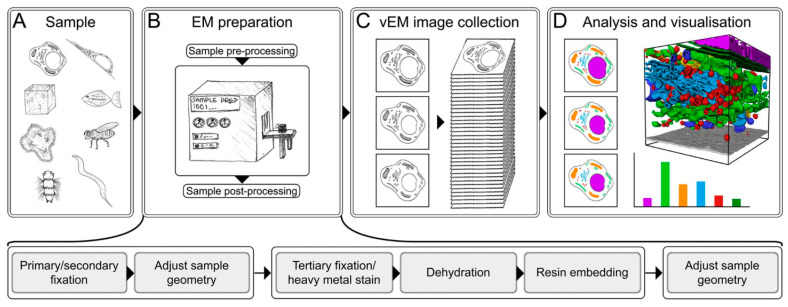
Experiment pipeline from [161] (CC BY 4.0). On the panel (**D**) organelles are highlighted by different colors.

Two volume electron microscopy methods, evolved from SEM, are serial block face scanning electron microscopy (SBF-SEM) [152] and Focused Ion Beam Scanning Electron Microscopy (FIB-SEM) [153]. Both methods generate a series of images (in SBF-SEM, the sample is sliced by microtome, in FIB-SEM by focused ion beam) that are stacked to form a volume digital representation of the original sample (Figure 6). Crucially, for research, both technologies enable a correlation with fluorescence light microscopy (three-dimensional correlative light and electron microscopy, 3D-CLEM), facilitating the identification of fluorescently labeled organelles or proteins of interest in the images. Moreover, FIB-SEM has been combined with super-resolution fluorescence microscopy such as interferometric PALM, visualizing the mitochondrial outer membrane via the immunolabeling of TOM20 with fluorescent tags [154]. Each method will be described in more detail below.

SBF-SEM is an effective tool for the analysis of the mitochondria density, size, and connectivity, and can even access the cristae-free volume, but it still should be supplemented by TEM techniques for a detailed analysis of the cristae morphology [155]. It was shown that such volumetric parameters of mitochondria obtained by SBF-SEM as the volume, shape, and distribution in the cell, including contacts with other organelles, are more sensitive to pathological changes (e.g., during injury/neurodegeneration) than the classical 2D-EM morphometry [156]. Importantly, the obtained serial slices of the sample can be subjected to histochemical staining, e.g., for cytochrome c oxidase activity, enabling the structural and functional correlations of mitochondria in tissue [157]. That is a very strong additional advantage of SBF-SEM.

The FIB-SEM technique involves the sequential removal of thin sample layers (down to several nanometers) and the scanning of the freshly exposed surface with an electron microscope. This alternating milling and imaging yield a continuous 3D image volume with an approximately 4 nm resolution isotropic across all axes. Compared to electron tomography or serial sectioning, FIB-SEM offers a more isotropic resolution. The advantages of modern implementations include the automation of scanning and the ability to examine very large tissue volumes (up to cubic millimeters), albeit with long acquisition times scaling with the volume size.

**Figure 7:**
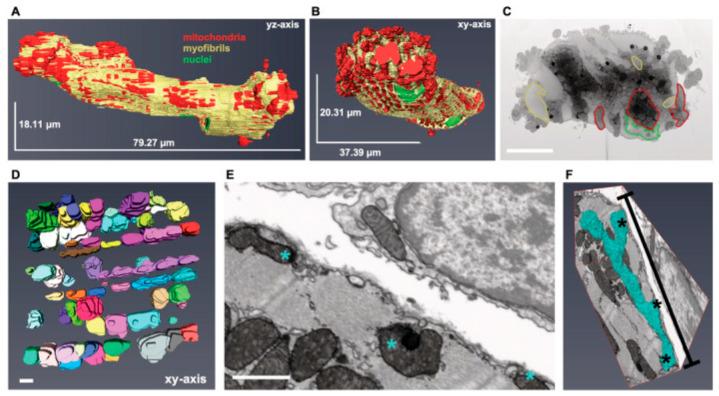
Three-dimensional (3D) mapping of mitochondria in isolated cardiomyocytes and cardiac tissue. (**A**,**B**) 3D reconstructions of a longitudinally (**A**) and transversally (**B**) oriented isolated mouse cardiomyocyte with mitochondria highlighted in red, myofibrils in yellow, and nuclei in green. (**C**) Electron micrograph of an isolated cardiomyocyte in a transverse orientation. Scale bar, 5 μm. (**D**) 3D reconstruction of the cardiomyocyte mitochondrial network in cardiac tissue (n = 3 wild-type mice, each with four z-stacks). Each mitochondrion is represented by a different color. Scale bar, 1 μm. (**E**,**F**) Two-dimensional (2D) image depicting three separate mitochondria ((**E**), asterisks). Note that the 3D reconstruction reveals merely one complex mitochondrion up to 6 μm with branches (indicated by *) ((**F**), cyan). Scale bar, 1 μm (1 wild-type mouse, z-stack). The figure from Heinen-Weiler et al. [158] (CC BY-NC-ND 4.0 license; panels (**A**–**F**) were used without changes). Refer to original article for animations and additional details.

In mitochondrial research, FIB-SEM enables highly detailed 3D reconstructions of mitochondrial networks and inner membrane structures, as well as studies of the organelle contacts and the detection of structural abnormalities under pathological conditions [158]. While other techniques can accomplish similar analyses, the lack of a volumetric context in those approaches may lead to incorrect conclusions. For example, mitochondria appearing as isolated entities on a single section may actually be interconnected within a mitochondrial network (see Figure 7).

Automated segmentation methods utilizing machine learning are actively being integrated for volumetric microscopy data analysis, as the manual processing of large datasets is practically infeasible [159,160].

Due to its rapid advancement and high resolution, the volumetric methods of scanning electron microscopy may, in the future, compete with super-resolution fluorescence techniques for the analysis of the mitochondrial networks and organelle structure. They enable an analysis of the mitochondrial morphology and size, and the packing characteristics of the inner membrane, which also serve as important diagnostic parameters [162]. The crucial limitation of these techniques is the pretreatment of the sample which somehow may disrupt the natural structure of tissue.

## 6. Scanning Probe Microscopy

**Figure 8:**
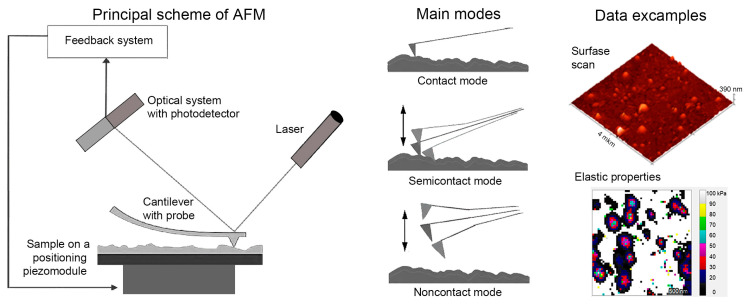
Scheme of mitochondria AFM study.

Scanning probe microscopy (SPM) encompasses a family of high-resolution techniques that employ physical interactions between a sharp probe and the sample surface to obtain topographical and mechanical information at the nano- and microscale levels. Among SPM methods, atomic force microscopy (AFM) is the most widely used for biological applications, allowing the visualization of the surfaces of both fixed and live cells or isolated organelles, as well as their examination under physiological conditions (Figure 8). Modern instruments support the high-speed imaging and integration of AFM with fluorescence microscopy [163]. Additionally, AFM provides insights into the mechanical properties of samples, which are inaccessible to optical or electron microscopy. Certain AFM modifications are gentle enough to monitor even self-assembly reactions driven by weak interactions [164].

### 6.1. Atomic Force Microscopy

Atomic force microscopy (AFM) has been successfully utilized for the visualization of the whole mitochondria [165] and mitoplasts [166], providing detailed information on the organelle sizes and membrane topography. Aldehyde fixation, for example, preserves the mitochondrial dimensions and cristae morphology after sample drying [167]. Using AFM, the morphological abnormalities of mitochondria were detected in isolated organelles under conditions such as alpha-synuclein overexpression [168]. AFM also permits the examination of sections of fixed cells or tissues, enabling the observation of morphological changes, for instance, from the normal state to swelling during apoptosis [169]. The use of any fixation method ensures structural stability.

The live imaging of mitochondria in a physiological buffer presents a greater technical challenge. The primary issue is that the AFM cantilever can collide with the sample during scanning, risking damage to both the probe and biological specimen. The AFM resolution in liquid is significantly reduced compared to air (for fixed and dried samples) due to the hydrodynamic damping of cantilever oscillations and thermal noise. Additionally, the resolution depends strongly on the sample fixation quality on the substrate [170]. The reduced mitochondrial mobility during imaging can be achieved by exploiting electrostatic forces through positively charged surfaces or surface treatments such as a polylysine coating. For example, a sufficiently high resolution to identify ATP synthase dimers was obtained on mitochondrial inner membrane samples adhered to mica surfaces [128].

The cell and organelle activity, including mitochondrial function, can be probed in a nontrivial manner using a sensor based on an AFM cantilever by placing the object on the cantilever rather than scanning with it. Mitochondrial activity alters the cantilever oscillations, thereby providing a functional readout [171]. Another approach for assessing the mitochondrial function uses AFM in tapping mode [172], where mitochondrial oscillations correlate with the membrane potential.

### 6.2. Use of the Young’s Modulus for Subsurface Structure Assessment

A promising approach for the indirect evaluation of subsurface structures involves the measurement of the elastic modulus (Young’s modulus) via AFM indentation. This method is based on the premise that the mechanical properties of the cell surface reflect the arrangement and state of the intracellular components, including mitochondria. Recent studies demonstrated the noninvasive mechano-functional analysis of individual liver mitochondria using AFM. This approach generates stiffness distribution maps at the nanometer resolution, correlating with the cristae and internal structures. However, it remains an indirect technique requiring complex mathematical approximations for data interpretation, limiting its applicability compared to direct visualization methods such as electron tomography.

### 6.3. Scanning Ion Conductance and Electrochemical Microscopy

Scanning ion conductance microscopy (SICM) offers an alternative to SPM by addressing adhesion issues through a fully non-contact operation mode. SICM employs a nanopipette filled with electrolyte and measures the ion current for the feedback control of the probe-sample distance. A principal limitation is the comparatively lower resolution in liquid relative to AFM and especially electron microscopy. SICM is suitable for imaging metabolically active organelles with lateral resolutions of approximately 60–150 nm and a vertical resolution around 1 nm [173].

Scanning electrochemical microscopy (SECM) shares operational similarity with SICM but uses a micropipette incorporating an electrode capable of sensing chemical processes such as the oxygen concentration and reactive oxygen species levels. Micropipettes may also serve for injections or localized air pressure application [174]. However, the application of these methods to isolated mitochondria is hindered by their small size, and their use on whole cells does not permit specific mitochondrial examination due to the probe placement solely on the cell surface. Given the mitochondrial similarities to bacteria in origin, size, and function, scanning electrochemical microscopy techniques proposed for bacterial studies may be applicable to mitochondria [175].

In summary, scanning probe microscopy—particularly AFM—is broadly applicable for mitochondrial studies across organizational scales, from whole organelles to isolated protein complexes. This method is advantageous for investigating living cells and mitochondria. Nevertheless, live mitochondrial visualization under physiological conditions remains technically challenging with significant resolution limitations. Alternative approaches such as SICM and Young’s modulus measurements of subsurface structures do not fully resolve these challenges.

## 7. X-Ray and Neutron Scattering Methods

### 7.1. Wide-Angle X-Ray Scattering

Similar to other biological specimens, wide-angle X-ray scattering is well-suited for investigating isolated and crystallized structures. Numerous mitochondrial protein structures have been elucidated using this method, including the earliest structures of ATP synthase subunits [176]. Since key proteins of the mitochondrial oxidative phosphorylation system are multi-component membrane complexes, their crystallization is challenging and can lead to structural artifacts. Consequently, cryo-electron microscopy has become a more practical method for resolving their structures. Nevertheless, crystallization remains relevant for studying smaller mitochondrial matrix proteins. Additionally, crystallization can provide insights into tightly associated lipid molecules bound to proteins, as exemplified by the c-ring of ATP synthase [177].

### 7.2. Small-Angle Neutron and X-Ray Scattering

Small-angle scattering (SAS) provides information on larger diffracting structures than individual proteins and can be applied to entire mitochondria. In the 1970s, small-angle X-ray scattering was employed to investigate intact mitochondrial structures [178], and, in the 2000s, analogous experiments using neutron scattering demonstrated comparable results [179]. Briefly, both methods detected diffraction changes from mitochondrial cristae associated with morphological alterations such as swelling or de-energization, correlating well with the electron microscopy data [180]. Due to the greater informativeness of electron microscopy, the SAS of whole mitochondria has seen limited application. However, SAS remains valuable as a complementary approach for assessing the size and shape of proteins in solution, including mitochondrial proteins [181].

### 7.3. X-Ray Tomography

**Figure 9:**
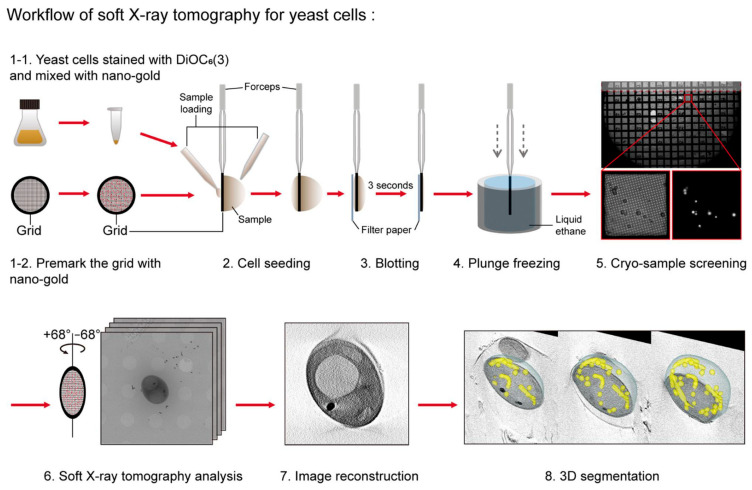
Brief experimental protocol for freezing yeast cell samples for subsequent X-ray tomography. From [183] (CC 4 license).

X-ray tomography has also been applied to mitochondrial studies [182]. Sample preparation for this method resembles cryo-electron microscopy, involving rapid freezing in liquid ethane or propane on grids or thin capillaries (Figure 9). X-ray methods enable the imaging of thicker samples compared to transmission electron microscopy, allowing the visualization of mitochondrial networks [183]. However, the spatial resolution is lower than that of cryo-electron tomography, reaching a maximum of approximately 35 nm, sufficient to reconstruct the overall shape of mitochondrial cristae but insufficient to resolve the individual protein localizations within them [184].

## 8. Spectroscopic Techniques

Spectroscopic methods differ in that they do not provide direct structural information but allow inference of interactions based on the dependence of a particular process’s intensity on the wavelength (e.g., absorption, reflection, fluorescence, luminescence, and scattering). This wavelength dependence is referred to as a spectrum, and its acquisition and analysis constitute spectroscopy. Consequently, spectroscopy encompasses a vast array of techniques founded on diverse physical phenomena. Often, spectroscopy enables the characterization of the sample structure or specific components thereof. Below, key spectroscopic methodologies applicable to mitochondria are elaborated, highlighting the structural and functional mitochondrial parameters they can assess.

### 8.1. Raman (Inelastic) Scattering Spectroscopy of Mitochondria

**Figure 10:**
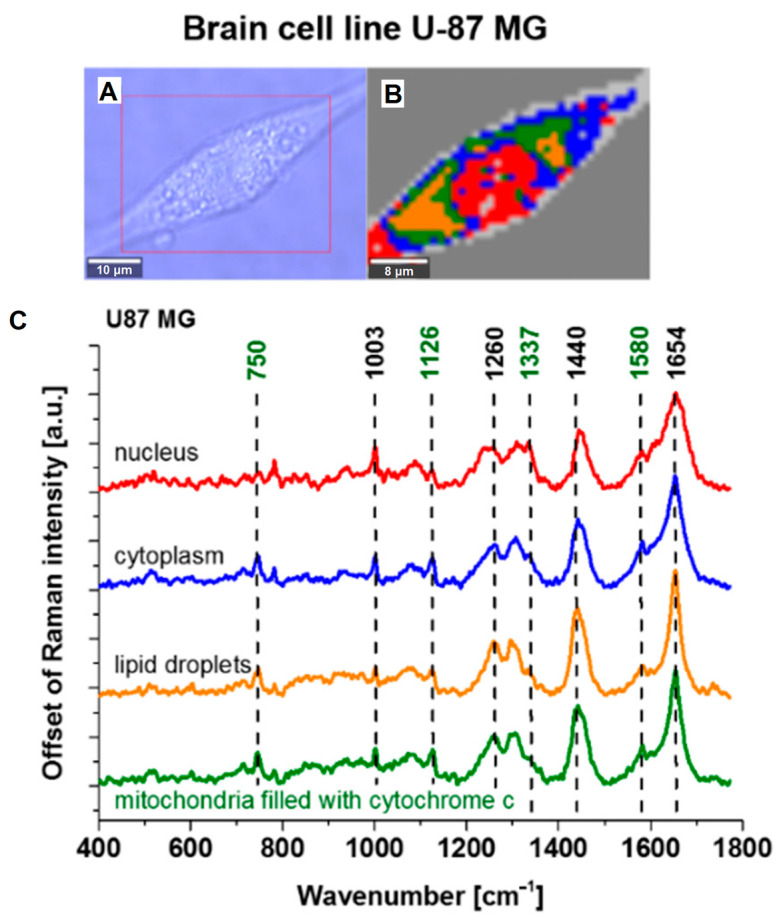
Image modified from [189]. (**A**) Raman image (40 μm × 30 μm, resolution 0.5 μm, integration time 1.0 s); (**B**) the average Raman spectra (number of cells n = 3, number of single Raman spectra n = 14,400) of nuclei (red), cytoplasm (blue), lipid droplets (orange), and mitochondria (green); and (**C**) at 532 nm of single-cell U-87 MG.

Raman spectroscopy relies on the inelastic scattering of photons. Primarily, light scatters elastically on molecules without a frequency change; however, a small fraction of photons undergo frequency shifts, resulting in additional lines in the scattering spectrum. An analysis of these lines, arising from the interaction of laser radiation with atomic vibrations in the sample, provides information about the vibrational modes of the system under study. This method is advantageous as it permits the investigation of functioning mitochondria using only laser irradiation without requiring labels. Certain Raman bands correspond specifically to vibrations of particular atomic groups. Raman spectroscopy is historically well-established and has been employed in biology, for example, to study cells and chromosomes [185]. It is also used to examine cytochrome-containing mitochondrial enzymes, whose Raman scattering depends on the oxidation state of the heme iron ion [186]. This technique facilitates an assessment of the mitochondrial metabolic activity via the reduction states of cytochromes (Figure 10) [187].

One challenge of Raman spectroscopy is the need for sufficiently intense signals, which has somewhat limited its application. Advances in technology and the implementation of signal enhancement strategies, such as surface-enhanced Raman spectroscopy (SERS) leveraging plasmon resonance, have improved its efficacy in cellular studies [188]. Raman spectroscopy enables spatial mapping by selectively irradiating different cellular regions, including areas with signals from mitochondrial cytochromes, thereby allowing a functional analysis of mitochondria based on their spectra [189]. An intriguing recent application is the discovery of a direct correlation between the conformational changes in cytochrome c heme and the membrane potential of functioning mitochondria [190], enabling the membrane potential assessment via Raman spectroscopy without the use of dyes.

An additional potential advancement of the method involves the use of entangled photons, which enhances the signal-to-noise ratio and thereby increases the sensitivity of biospectroscopic investigations [191], as well as potentially providing novel information about complex molecules or their assemblies [192]. To the authors’ knowledge, such an approach has not yet been applied to mitochondria or mitochondrial proteins.

Overall, Raman spectroscopy constitutes a highly powerful biophysical technique that can effectively monitor the redox states of all cytochromes, depending on the laser excitation wavelength employed. Despite this, its application remains relatively limited compared to more conventional biochemical and fluorescence methodologies. The limited adoption may partly stem from the complexity of spectral analysis for biologists or the lack of the widespread availability of the necessary instrumentation. Nevertheless, there are emerging examples of the method’s use for obtaining clinically relevant data, such as predicting sepsis development [193], detecting circulating cancer cells [194], and identifying mitochondrial dysfunction in neurodegeneration [195].

### 8.2. Nuclear Magnetic Resonance (NMR)

NMR enables metabolic profiling in tissues as well as in isolated mitochondria. Information regarding mitochondrial metabolites can be obtained via magic-angle spinning NMR, which mitigates the contributions from intermolecular interactions. However, a higher resolution, necessary for the detection of lower metabolite concentrations, requires more complex protocols involving the preliminary extraction of metabolites from isolated mitochondria [196]. This approach allows an investigation of tissue-specific mitochondrial metabolite distributions and the monitoring of changes in pathological conditions, including cancer [196].

Isotope tracer-based NMR spectroscopy constitutes another interesting approach, wherein isotope-labeled metabolites are introduced into cells or mitochondria, and real-time metabolism is tracked by monitoring isotope incorporation into downstream compounds [197,198]. In the mitochondrial context, this technique also facilitates monitoring tissue oxygen concentrations using NMR via the spectra of perfluorocarbons added to the sample, which bind oxygen [199]. The combination of NMR with other methods, such as near-infrared spectroscopy, allows the measurement of tissue ATP/O_2_ ratios [200], a quantitative indicator of oxidative phosphorylation efficiency.

Phosphorus NMR (^31^P-NMR) studies of whole mitochondria further permit the assessment of phospholipid packaging and mobility [201,202]. This method demonstrated the presence of non-bilayer, protein-immobilized lipids in mitochondria, correlating their an abundance with ATP synthesis rates [203]. ^31^P-NMR has even been employed to measure mitochondrial ΔpH based on changes in phosphate group signals [204].

NMR is also instrumental in addressing various questions indirectly related to mitochondria using isolated proteins or lipids. For example, NMR determined the pK of a key mitochondrial lipid, cardiolipin [205], and revealed strong interactions between cardiolipin and certain inner membrane proteins [206]. Additionally, NMR is a crucial technique for studying the protein structure, including mitochondrial proteins, providing complementary structural and dynamic information spanning the atomic to entire protein molecule levels [207]. Comprehensive descriptions of NMR capabilities, illustrated by studies of Fe-S clusters, are detailed in specialist reviews such as Cai and Markley [208].

Thus, NMR represents one of the most powerful physical methods for investigating tissues, intact mitochondria, and their components, including isolated proteins, lipids, and extracted metabolites.

## 9. Method Selection Framework: Integrating Approaches for Mitochondrial Research

**Figure 11:**
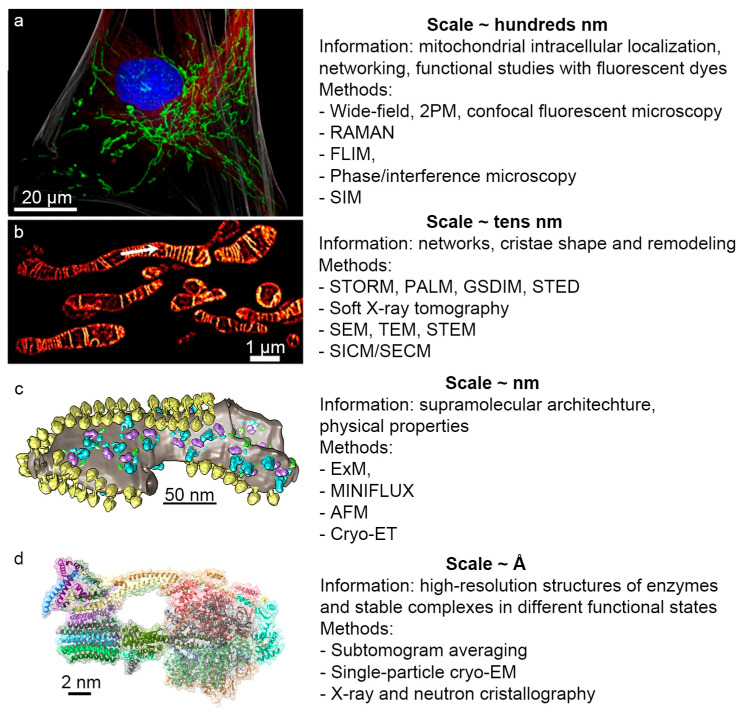
Methods for studying mitochondria at different scales and selected examples of the images obtained. (**a**) Confocal image of chemically fixed human skin fibroblast. The mitochondrial network of a primary fibroblast with labeled mitofilin (green), microtubule cytoskeleton (red), nucleus (blue), and actin cytoskeleton (gray). Modified from [209]. (**b**) STED imaging of mitochondria of living HeLa cells stained with MitoPB Yellow after deconvolution. Modified from [210]. (**c**) Supramolecular architecture of mitochondrial cristae membrane with reconstructed respiratory chain complexes (I—blue, III—magenta, and IV—green) and ATP synthases (yellow) [131]. (**d**) 2.8 Å-resolution structure of human ATP synthase obtained by cryo-TEM (pdb 8H9T) [211].

The diversity of available methods for investigating the mitochondrial structure and function presents both opportunities and challenges for researchers. While the preceding sections have described each method category in detail, selecting the optimal approach for a specific research question requires careful consideration of multiple factors including the biological question, sample type, required resolution, and practical constraints. This section provides an integrated framework for method selection, organizing the techniques according to the research objectives they address most effectively while explicitly discussing their complementary relationships and relative trade-offs. The quantitative assessment of the mitochondrial morphology represents one of the most common applications of mitochondrial imaging, providing integrative indicators of the organelle functional state. The choice of method depends critically on whether the study requires live-cell imaging, the level of spatial detail needed, and whether functional parameters such as the membrane potential must be assessed simultaneously. The brief information about the key discussed methods is summarized in Table 2 and Figure 11.

Fluorescence-based approaches commonly allow live-cell imaging and structure-functional study, while their common limitations include invasiveness, phototoxicity, probe imperfections (photobleaching, washout, and efflux by transporters), and the need for cell fixation and use of special dyes for some methods. These methods have a wide resolution progression for almost any task. The order is as follows: confocal (250 nm) → SIM (100 nm) → STED (40–50 nm, cristae-level) → STORM/PALM/GSDIM (10–30 nm, but fixed samples) → MINFLUX (1–5 nm, sophisticated and expensive). 2PM is a must-have technology if in-tissue visualization is needed. ExM is also a special case, making super-resolution accessible on conventional microscopes, but with poorly-controlled artifacts caused by hydrogel anchoring and stretching. The acquired information can be further complemented by FLIM-based techniques.

One of the modern innovations is the emergence of dye-free techniques for detecting mitochondria in a cell. Phase microscopy allows label-free network visualization (with machine pre-learning on fluorescent data, which may cause inaccuracies) and has no phototoxicity, but is still diffraction-limited. Raman spectroscopy provides mitochondria localization based on metabolic/redox information, and also, with additional complications. may estimate the membrane potential via the cytochrome c state. This is a promising, but not yet widely available technique. The FLIM of endogenous NADH allows probe-free metabolic profiling with two-photon depth penetration. It is also a state-of-the-art technique awaiting greater adoption.

Electron microscopy provides a greater level of detail, but completely eliminates the observation of a living cell. TEM is a gold standard for cristae visualization and it is still relevant as a result of the proven technology and fast throughput. It has poorly studied fixation artifacts and gives limited 3D info. The latter disadvantage is corrected by the additional use of scanning microscopy, in combination with TEM (TSEM techniques) or separately. Volume EM (FIB-SEM/SBF-SEM) is ideal for the visualization of the 3D networks and ultrastructure at 4–10 nm over large volumes. The main limitations are long acquisition times and fixed samples with special pretreatment. X-ray tomography, if available, can be an alternative for the visualization of large volumes, but usually has a lower resolution than volume EM.

Cryo-ET offers more native structure preservation, with enough resolution for protein complex visualization in the membrane or matrix context. But it has thickness constraints and an even lower throughput. One of the greatest strong points of cryo-ET is the possibility to carry out subtomographic averaging to achieve the resolution of the protein complex structure close to single-particle cryo-TEM and even X-ray cristallography, but in the native environment of the protein. Nevertheless, if it is necessary to obtain a high-resolution protein structure, the latter two methods are still preferable. It should be noted that additional data about the protein structure and inter- and intramolecular interactions can be obtained using NMR, Raman spectroscopy, small-angle neutron, and X-rays diffraction. In this aspect, mitochondrial proteins are no different from the rest.

It is important to note that electron microscopy is very compatible with fluorescent-based techniques, as a result of the development of technology and the advent of systems that make it possible to make CLEM. Depending on the technical equipment, EM can be combined with both traditional widefield microscopy and some super-resolution techniques.

For several methods, machine learning is an integral part. It has two sides now—first, it allows analyzing much larger data sets and decreasing person-based discrepancies. On the other hand, it critically depends on the initial training data set (the annotation quality and instrument in which it was obtained) that may impede the free transfer of pre-trained models between laboratories, reduce the reproducibility of results, and lead to unintentional distortions. For small samples or those with significant peculiarities, it is still more reasonable to use manual processing by specialists. At the same time, many machine-learning-based approaches have already proven themselves to be indispensable components of data processing (for example, for cryo-TEM). Development in this area, optimized for standard instrumentation, will likely mitigate most of these shortcomings in the future.

The methods described in this review should not be viewed as alternatives but, rather, as complementary tools that, when combined strategically, enable the comprehensive characterization of mitochondria across organizational levels. CLEM bridges these scales by precisely correlating functional fluorescence signals with ultrastructural features, while additional methods (Raman, NMR, and SPM) integrate metabolic/mechanistic information that imaging alone cannot provide.

## 10. Conclusions

The advancements in mitochondrial research methods have substantially expanded the scope of the structural and functional analysis of these organelles, enabling high-resolution investigations into their dynamics, morphology, and bioenergetic properties. Modern imaging technologies such as super-resolution microscopy, electron tomography, and integrative correlative approaches now allow researchers to study the mitochondrial ultrastructure in living cells with unprecedented detail and precision in almost any scale (Figure 11). The combination of staining agents, genetically encoded indicators, and machine-learning-based image analysis has provided tools to quantitatively assess the mitochondrial function across diverse biological contexts and disease models.

Overall, the integration of these state-of-the-art techniques in conjunction with robust computational and analytical frameworks has propelled mitochondrial science into a new era, fostering discoveries in cellular physiology, metabolic regulation, oxidative stress response, and mitochondrial involvement in pathology. The continuous evolution of methodological approaches is expected to further facilitate breakthroughs in understanding the complex roles of mitochondria in health and disease.

## Figures and Tables

**Table 1:** Properties of the mitochondria-targeted dyes. Notes: Ratiometric probes (JC-1, DiOC2(3), and Mito-pH) provide emission shifts that enable quantitative measurements independent of dye concentration. Fixability indicates whether the dye remains bound after aldehyde fixation and permeabilization. ‘Partial’ fixability indicates variable retention depending on fixation protocol. ROS probes (MitoSOX Red, MitoPY1, MitoAR, and MitoHR) require membrane potential for mitochondrial uptake but report on specific reactive species rather than potential directly.

Dye	Ex/Em (nm)	Membrane Potential Dependence	Fixability	Working Concentration
MitoTracker Green FM	490/516	No	Partial	50–500 nM
MitoTracker Red CMXRos	578/599	Yes	Yes	25–200 nM
MitoTracker Deep Red FM	644/665	No	Partial	50–200 nM
TMRM	548/573	Yes	No	1–30 nM
TMRE	549/574	Yes	No	10–100 nM
Rhodamine 123	505/534	Yes	No	1–10 μg/mL
JC-1	485/530 535/590	Yes (ratiometric)	No	1–10 μM
DiOC2(3)	482/497	Yes (ratiometric)	No	0.5–50 μM
MitoView 633	622/648	Yes	No	50–200 nM
HBmito Crimson	658/678–690	No (membrane-binding)	Partial	50–200 nM
PKMO (COT-Cy3)	584/604	No (protein label)	Yes	0.5–10 μM
MitoSOX Red and MitoHR	510/580	Yes (uptake)	No	100 nM–1 μM
MitoPY1	503–510/528	Yes (uptake)	No	1–10 μM
MitoAR	548/571	Yes (uptake)	No	1–5 μM
Mito-pH	490/520 560/600	Yes (uptake)	No	0.5–10 μM

**Table 2:** Key methods comparison.

Method	Resolution	Live Cell	Best for	Key Limitation	Throughput
Widefield and confocal microscopy	~250 nm	Yes	Network dynamics, functional parameters	Diffraction limited, artifacts of fluorescent dyes	High
Raman microscopy	~250 nm–1 µm	Limited	Spatial mapping of different cellular regions by their spectra, membrane potential estimation	Low signal, should be combined with other techniques, difficult analyses	High
FLIM	~250 nm	Yes	Mitochondrial metabolism, ultrastructure, and dynamics	Photobleaching, low signal-to-noise ratio, environment artifacts	Medium
2PM	~400 nm	Yes	Imaging with deep tissue penetration	Near-infrared wavelengths, scattering, focal volume photodamage	High
Phase/interference microscopy	~120–250 nm	Yes	Mitochondria in-cell localization and network dynamics without dyes	Needs machine-learning pretraining on fluorescent microscopy data	High
SIM	~100 nm	Yes	Network dynamics with improved resolution	Still cannot resolve single cristae	High
STED	40–50 nm	Yes	Cristae structure and dynamics	High photodamage risk	Medium
STORM/PALM/GSDIM	10–30 nm	Limited	Protein localization	Fixed samples preferred, use of special dyes	Low
ExM	10–70 nm	No	In cristae protein localization	Artifacts from hydrogel effects, non-isotropic expansion possibility	Low/Medium
MINFLUX	1–5 nm	Yes	Molecular assemblies	Complex instrumentation	Very Low
TEM/TSEM	1–2 nm	No	Cristae structure, in tissue localization	Fixation artifacts	Medium
Cryo-TSEM	1–2 nm	No	Cristae structure, in tissue localization	Poorly controlled structure damage under cell “unroofing”	Low
Cryo-ET	2–5 nm (<2 nm for averaging)	No	Cristae structure, organelle and protein interactions, protein structures	Sample thickness limit, surface effects, irradiation damaging	Low/Very Low
FIB-SEM and SBF-SEM	~4 nm	No	3D networks, cristae, large volumes	Long acquisition time, fixation artifacts	Very Low
SPM (AFM, SICM, SECM)	1–10 nm	Limited	Surface topology, mechanics, limited functional parameters	Surface only, isolated mitochondria, lower resolution for unfixed samples	Low
Single particle cryo-TEM	0.1–1 nm	No	Protein structure	Pure protein samples	Medium
X-ray crystallography	0.1–1 nm	No	Protein structure	Only crystallized proteins	Very Low
NMR	0.1–1 nm	No	Intraprotein structure, metabolomics	Pure proteins or tissue extracts (for metabolites)	Low

## Data Availability

No new data were created or analyzed in this study. Data sharing is not applicable to this article.

## Abbreviations

The following abbreviations are used in this manuscript:

2PM	two-photon microscopy
AFM	atomic force microscopy
CLEM	correlative light and electron microscopy
CLSM	confocal laser scanning microscopy
COT	cyclo-octatetraene
cryo-	cryogenic
DIOC2(3)	3,3′-dihexyloxacarbocyanine iodide
ED-FRAP	enzyme-dependent fluorescence recovery after photobleaching
EM	electron microscopy
ET	electron tomography
ExM	expansion microscopy
FIB-SEM	focused ion beam scanning electron microscopy
FLIM	fluorescence lifetime imaging microscopy
FCCP	carbonyl cyanide-p-trifluoromethoxyphenylhydrazone
FRAP	fluorescence recovery after photobleaching
GCaMP	genetically encoded calcium indicator (protein)
GECO	genetically encoded calcium indicator
GECIs	genetically encoded calcium indicators
JC-1	5,5′,6,6′-tetrachloro-1,1′,3,3′-tetraethylbenzimidazolylcarbocyanine iodide dye
MaLion	ATP biosensor (“Monitoring ATP Level intensiometric turn-on indicators”)
MICOS	mitochondrial contact site and cristae organizing system
MitoAR/MitoHR	mitochondria-targeted redox sensors
Mito-pH	genetically encoded mitochondrial pH sensor
MitoPY1	mitochondria-targeted hydrogen peroxide probe
MitoSOX Red	mitochondria-targeted superoxide indicator
MitoTracker Green FM	mitochondria dye (membrane potential independent)
MitoTracker Red CMXRos	mitochondria dye (reactive to thiols in proteins)
MitoTracker Deep Red FM	mitochondria dye (far-red emission with high photostability)
MitoView 633	mitochondria dye (far red, photostable)
MINFLUX	minimal emission fluxes localization microscopy
NADH-FLIM	nicotinamide adenine dinucleotide fluorescence lifetime imaging
NMR	nuclear magnetic resonance
OS-PCM	organelle-specific phase contrast microscopy
PALM	photoactivated localization microscopy
PKMO (COT-Cy3)	cyanine dye conjugated with cyclo-octatetraene
PSC833	P-glycoprotein inhibitor
ROS	reactive oxygen species
SAS	small-angle scattering
SBF-SEM	serial block face scanning electron microscopy
SECM	scanning electrochemical microscopy
SEM	scanning electron microscopy
SICM	scanning ion conductance microscopy
SIM	structured illumination microscopy
Si-rodamine	silicon-rhodamine dye
SPADE	start point-driven analysis (in image analysis)
SPM	scanning probe microscopy
STED	stimulated emission depletion microscopy
STEM	scanning transmission electron microscopy
STORM	stochastic optical reconstruction microscopy
TEM	transmission electron microscopy
TMRM	tetramethylrhodamine methyl ester
TMRE	tetramethylrhodamine ethyl ester
